# Expressions of shox2, RASSF1A and PTGER4, and the relationship between their methylation and clinicopathological characteristics in patients with lung cancer

**DOI:** 10.5937/jomb0-54540

**Published:** 2025-03-21

**Authors:** Haitao Su, Qiaoye Lv, Limei Wang, Jigang Hu, Yinggang Lv, Liting Jia, Qingbin Qi

**Affiliations:** 1 Affiliated Hospital of Hebei University of Technology, Department of Respiratory Medicine, Handan, China; 2 Affiliated Hospital of Hebei University of Technology, Department of Imaging, Handan, China; 3 Affiliated Hospital of Hebei University of Technology, Sterilization and Supply Center, Handan, China; 4 Affiliated Hospital of Hebei University of Technology, Department of Thoracic Surgery, Handan, China; 5 Affiliated Hospital of Hebei University of Technology, Department of Pathology, Handan, China

**Keywords:** lung cancer, short stature homobox2, rasassociation domain family 1A, prostaglandin E receptor 4, methylation, clinicopathology, rak pluća, homobok2 niskog rasta, porodični domen ras-asocijacije 1A, receptor prostaglandina E 4, metilacija, klinička patologija

## Abstract

**Background:**

To explore the expressions of short stature homobox2 (SHOX2), Ras-association domain family 1A (RASSF1A) and prostaglandin E receptor 4 (PTGER4), and the relationship between their methylation and clinicopathological characteristics in patients with lung cancer (LC).

**Methods:**

The surgical specimens of cancer tissues and para-carcinoma tissues were collected from 50 patients with LC in the Affiliated Hospital of Hebei University of Engineering between January and November 2023. The expressions of SHOX, RASSF1A and PTGER4 proteins in cancer tissues and para-carcinoma tissues were detected by immunohistochemistry, and methylation status of SHOX, RASSF1A and PTGER4 genes in peripheral venous blood was detected by sulfite-modified real-time fluorescence quantification. The positive expression rates of SHOX2, RASSF1A and PTGER4, and positive rates of SHOX2, RASSF1A and PTGER4 genes methylation in cancer tissues and para-carcinoma tissues were compared. The relationship between SHOX2, RASSF1A, PTGER4 methylation and clinicopathological characteristics in LC patients was compared by real-time fluorescence quantitative PCR.

**Results:**

The positive expression rates of SHOX2, RASSF1A and PTGER4 in cancer tissues were 44.0%, 54.00% and 50.00%, significantly lower than those in para-carcinoma tissues (90.00%, 96.00%, 82.00%, P<0.05). The methylation positive rates of SHOX2, RASSF1A and PTGER4 genes in cancer tissues were 48.00%, 32.00% and 64.00%, significantly higher than those in para-carcinoma tissues (16.00%, 4.0%, 14.00%, P<0.05). In terms of different pathological types and TNM staging, methylation positive rates of SHOX2 and RASSF1A genes were the highest in patients with adenocarcinoma and TNM staging at stage IV. However, since the study included a limited sample size and only two major histological subtypes of LC, caution is needed in generalizing these findings across all stages and types of LC. Larger, more diverse studies are needed to determine if these findings can be broadly applied to other LC subtypes and stages. In terms of different pathological types, methylation positive rate of PTGER4 gene was the highest in patients with TNM staging at stage IV (P<0.05). While these results indicate a correlation between PTGER4 methylation and advanced disease stage, further research with a larger, more heterogeneous patient population is necessary to validate whether these findings hold true across all stages and histological subtypes of LC.

**Conclusions:**

The low expressions and high methylation of SHOX2, RASSF1A and PTGER4 are common in LC patients. However, it is important to note that the small sample size and the cross-sectional nature of this study limit the ability to generalize these findings to a broader population. Additionally, the lack of longitudinal data means we cannot assess how methylation changes over time or in response to treatment, which is a significant limitation. The methylation positive rates of SHOX2 and RASSF1A genes are related to pathological types and TNM staging, and methylation positive rate of PTGER4 gene is only related to pathological types. These findings suggest that SHOX2, RASSF1A, and PTGER4 gene methylation could be valuable biomarkers for the early detection of LC, particularly in high-risk populations or those with atypical presentations. However, further studies with larger sample sizes and longitudinal designs are needed to confirm these findings and evaluate the long-term clinical utility of these methylation markers in monitoring disease progression and treatment response.

## Abstract (sr)

**Uvod:**

Cilj je bio da se istraži ekspresija homobok2 niskog rasta (SHOKS2), porodice domena Ras-asocijacije 1A (RASSF1A) i receptora prostaglandina E 4 (PTGER4), i odnos između njihove metilacije i kliničkopatoloških karakteristika kod pacijenata sa karcinomom pluća (LC).

**Metode:**

Hirurški uzorci tkiva raka i tkiva parakarcinoma prikupljeni su od 50 pacijenata sa LC u pridruženoj bolnici Hebei univerziteta inženjeringa između januara i novembra 2023. Ekspresije SHOKS, RASSF1A i PTGER4 proteina u tkivima raka i para-tkiva karcinoma su otkrivena imunohistohemijom i statusom metilacije SHOKS, RASSF1A i PTGER4 geni u perifernoj venskoj krvi detektovani su sulfitom modifikovanom kvantifikacijom fluorescencije u realnom vremenu. Upoređene su pozitivne stope ekspresije SHOKS2, RASSF1A i PTGER4 i pozitivne stope metilacije gena SHOKS2, RASSF1A i PTGER4 u tkivima raka i tkivima parakarcinoma. Odnos između metilacije SHOKS2, RASSF1A, PTGER4 i kliničko-patoloških karakteristika kod pacijenata sa LC upoređen je kvantitativnim PCR-om fluorescencije u realnom vremenu.

**Rezultati:**

Pozitivne stope ekspresije SHOKS2, RASSF1A i PTGER4 u tkivima raka bile su 44,0%, 54,00% i 50,00%, značajno niže od onih u tkivima parakarcinoma (90,00%, 96,00%, 82,00%, P<0,05). Pozitivne stope metilacije gena SHOKS2, RASSF1A i PTGER4 u tkivima raka bile su 48,00%, 32,00% i 64,00%, značajno veće od onih u tkivima parakarcinoma (16,00%, 4,0%, 14,00%, P<0,05). U pogledu različitih patoloških tipova i TNM stadijuma, pozitivne stope metilacije gena SHOKS2 i RASSF1A bile su najveće kod pacijenata sa adenokarcinomom i TNM stadijumom u IV stadijumu. Međutim, pošto je studija uključila ograničenu veličinu uzorka i samo dva glavna histološka podtipa LC, potreban je oprez u generalizaciji ovih nalaza u svim stadijumima i tipovima LC. Potrebne su veće, raznovrsnije studije da bi se utvrdilo da li se ovi nalazi mogu široko primeniti na druge podtipove i stadijume LC. U pogledu različitih patoloških tipova, stopa pozitivne metilacije PTGER4 gena bila je najveća kod pacijenata sa TNM stadijumom u IV stadijumu (P<0,05). Dok ovi rezultati ukazuju na korelaciju između metilacije PTGER4 i uznapredovale faze bolesti, neophodna su dalja istraživanja sa većom, heterogenijom populacijom pacijenata da bi se potvrdilo da li su ovi nalazi tačni u svim stadijumima i histološkim podtipovima LC.

**Zaključak:**

Niska ekspresija i visoka metilacija SHOKS2, RASSF1A i PTGER4 su uobičajeni kod pacijenata sa LC. Međutim, važno je napomenuti da mala veličina uzorka i priroda poprečnog preseka ove studije ograničavaju mogućnost generalizacije ovih nalaza na širu populaciju. Pored toga, nedostatak longitudinalnih podataka znači da ne možemo proceniti kako se metilacija menja tokom vremena ili kao odgovor na tretman, što je značajno ograničenje. Pozitivne stope metilacije gena SHOKS2 i RASSF1A povezane su sa patološkim tipovima i TNM stadijumom, a pozitivna stopa metilacije gena PTGER4 povezana je samo sa patološkim tipovima. Ovi nalazi sugerišu da bi metilacija gena SHOKS2, RASSF1A i PTGER4 mogli biti vredni biomarkeri za rano otkrivanje LC, posebno u populacijama visokog rizika ili onima sa atipičnim prezentacijama. Međutim, potrebne su dalje studije sa većim uzorcima i longitudinalnim dizajnom da bi se potvrdili ovi nalazi i procenila dugoročna klinička korisnost ovih markera metilacije u praćenju progresije bolesti i odgovora na lečenje.

## Introduction

Lung cancer (LC) is one of the malignant tumours with the highest morbidity and mortality rates worldwide, and patients are already in the middle and late stages of incurable disease at the time of diagnosis, with a very low 5-year survival rate, which poses a serious threat to human health and life safety [1]. In the past, clinical screening for early detection of LC typically involved tumour markers, bronchoscopy, cytology, and imaging. However, these methods were limited because early-stage symptoms are often atypical, and diagnosis mainly relied on clinicopathological features [2]. As epigenetics research in LC has advanced, the role of DNA methylation—beyond gene nucleotide sequences—has gained increasing attention for its impact on the pathogenesis and progression of LC [3]. Hypermethylation can lead to abnormal activation of oncogenes, causing chromosomal instability, which plays a key role in tumour development. When compared with other biomarkers, gene methylation, including that of SHOX2, RASSF1A, and PTGER4, could complement existing diagnostic tools. However, the sensitivity and specificity of these methylation markers, especially in earlystage LC, must be validated through larger and longitudinal studies before they can be integrated into routine clinical practice [4], so it is of positive significanceto screen LC early in the clinic by detecting the degree of gene methylation. Short stature homobox2 (SHOX2) is an important oncogene in the homobox family, which is usually involved in the control of cell differentiation in the form of regulating gene expression, and its methylation can lead to the silencing of oncogenic function and contribute to the formation of cancers [5]. Ras-association domain family 1A (RASSF1) is an important oncogene in the family of homoboxes, family 1A (RASSF1A) is a novel tumour oncogene cloned from the short arm of human chromosome 3, and its high methylation is highly correlated with the degree of differentiation of malignant tumours, which is often predictive of low patient survival [6]. Prostaglandin E receptor 4 (PTGER4) is an important oncogene of the G protein-coupled family, and its high or low gene methylation is often used as a reliable indicator to monitor the efficacy of LC [7]. In recent years, some studies have reported the value of SHOX2 and RASSF1A gene methylation in the clinicopathological diagnosis of lung cancer [6], while there are not many studies on the expression of SHOX2, RASSF1A, and PTGER4 and their methylation under different clinicopathological features in lung cancer patients. In this study, we mainly investigated and analysed the relationship between the expression of SHOX2, RASSF1A, PTGER4 and their methylation and the clinicopathological features of LC patients, with a view to providing a realistic and feasible reference basis for the clinical non-invasive examination of LC.

## Materials and methods

### General information

Surgical specimens of cancerous tissue and paracancerous tissue 3 cm from the edge of the cancerous lesion) of 50 LC patients admitted to the Affiliated Hospital of Hebei University of Engineering from January 2023 to November 2023 were selected. The sample size of 50 was determined based on feasibility, given the number of eligible patients available during the study period. While this sample size was adequate for an exploratory study, we acknowledge that a larger cohort would improve statistical power and strengthen the significance of the results.There were 34 males and 16 females among the 50 LC patients with the age ranging from 39 to 82 years old; the average age was (61.78±4.26) years old; the type of tumour was: squamous carcinoma in 39 cases, and adenocarcinoma in 11 cases; the degree of differentiation: highly differentiated in 9 cases, moderately differentiated in 31 cases, and lowly differentiated in 10 cases; the TNM stage: stage I in 12 cases, stage II in 28 cases, stage III in 10 cases. Degree of differentiation: 9 cases of highly differentiated, 31 cases of moderately differentiated, 10 cases of lowly differentiated; TNM staging: 12 cases of stage I, 28 cases of stage II, 10 cases of stage III. Inclusion criteria: meeting the diagnostic criteria for LC in the Chinese Medical Association Clinical Diagnosis and Treatment Guidelines for Lung Cancer (2022 edition) [8]; all underwent radical surgery and obtained cancer and paracancerous tissue specimens in our hospital; complete clinical data. Exclusion criteria: preoperative systemic chemotherapy, local radiotherapy and other anti-tumour treatments; the presence of active infection or central nervous system metastasis; and the combination of other malignant tumours or psychiatric and psychological diseases. The study was approved by the Ethics Committee of the Affiliated Hospital of Hebei University of Engineering, and all patients signed an informed consent form.

### Method

### Collection of clinical pathological characteristic data

Data on clinicopathological characteristics such as age, gender, smoking history, pathological type (adenocarcinoma or phosphocarcinoma), tissue differentiation (highly differentiated, moderately differentiated, or poorly differentiated), maximum diameter of tumour, TNM stage (Stage II, Stage III, or Stage IV), and metastasis to lymph nodes were collected from 50 patients with LC through the hospital’s electronic medical record system. We acknowledge that smoking history and genetic predisposition were not statistically adjusted for in the primary analysis due to sample size limitations. However, we have conducted subgroup analyses based on smoking history and other clinical characteristics to explore their potential impacts on gene expression and methylation.

### SHOX, RASSF1A, and PTGER4 protein expression detection and result determination

Lung cancer tissues and paracarcinoma tissues were fixed in 10% neutral formaldehyde solution and preserved as paraffin specimens. The paraffin specimens were routinely deparaffinised, endogenous peroxidase was blocked by 3% methanol H_2_O_2_, primary antibody, biotinylated secondary antibody and horseradish peroxidase-labelled tertiary antibody were added sequentially, the nuclei of the cells were stained with hematoxylin after DAB, and the slices were routinely dehydrated until transparent and then sealed with neutral gum. 10 randomly selected sections were taken from each section and the number of positive cells in each field of 100 cells were counted and averaged. high magnification fields of view, and the number of positive cells in 100 cells counted in each field of view was taken as the mean value. SHOX2, RASSF1A, and PTGER4 positive expression was defined by the combined score of positive cell count and staining intensity, with a total score ≥4 considered as positive. The positive cell count score was categorized as follows: 0–10% (score 0), 11%–25% (score 1), 26%–50% (score 2), 51%–75% (score 3), and 76%–100% (score 4). The staining intensity score was rated as: 0 for no staining, 1 for pale yellow, 2 for tan, and 3 for brown [9]. Positive cell count score of 0–10% was 0, 11%–25% was 1, 26%–50% was 2, 51%–75% was 3, and 76%–100% was 4. Staining intensity score was 0 for no staining, 1 for pale yellow, 2 for tan, and 3 for brown.

### Methylation detection and result determination of SHOX, RASSF1A, and PTGER4 genes

Take 10 mm paraffin specimens to make 10~20 slices, and routinely deparaffinize them. The DNA was extracted using the supporting DNA extraction kit and then reconverted by sulfite to convert unmethylated cytosine to uracil. The detection of methylation of DNA samples of SHOX, RASSF1A and PTGER4 was detected by using the real-time fluorescence quantitative PCR instrument model 7500 of ABI Company of the U.S.A. using specific primers combined with the fluorescent probe technology, and the quality control product was selected, the inflection point of the amplification curve was determined according to the actual amplification curve, and the position of the threshold line was adjusted to obtain the SHOX, RASSF1A and RASSF1A FAM, CY5 fluorescence signal Ct values. When enough DNA was added, the CY5 of the internal reference gene had a signal and the Ct value was 18 Ct 23, which indicated that the experiment was reliable and the DNA concentration was appropriate, and the FAM results could be read. SHOX, RASSF1A and PTGER4 were all judged to be positive for methylation with a FAM Ct value of <31. This threshold was used to determine the methylation positivity, as FAM Ct values below 31 indicate reliable methylation detection [10].

### Observation indicators

(1) To compare the positive expression rates of SHOX2, RASSF1A, and PTGER4 in cancer tissues and paracancerous tissues of LC patients; (2) To compare the positive rates of SHOX2, RASSF1A, and PTGER4 gene methylation in cancer tissues and paracancerous tissues of LC patients; and (3) To compare the real-time fluorescence quantitative PCR assay for the detection of SHOX2, RASSF1A, and PTGER4 methylation with clinicopathological features of LC patients.

### Statistical processing

The data were analyzed using SPSS 22.0 statistical software, and the measurement data wereexpressed as (x̄ ± s), and the differences were tested by t-test, and the count data were expressed as %, and the differences were tested by χ^2^ test or continuous corrected chi-square test; P < 0.05 was considered as statistically significant difference. However, given the relatively small sample size, the results should be interpreted with caution, and future studies with larger sample sizes are recommended to validate the findings and ensure more robust statistical power.

## Results

### Comparison of SHOX2, RASSF1A, and PTGER4 expression in cancerous and paracancerous tissues of LC patients

The positive expression rates of SHOX2, RASSF1A, and PTGER4 in cancer tissues of LC patients were 44.0%, 54.00%, and 50.00%, respectively, which were significantly lower than those in paracancerous tissues, which were 90.00%, 96.00%, and 82.00%, respectively (P < 0.05). See Table 1.

**Table 1:** Comparison of the expression of SHOX2, RASSF1A, and PTGER4 in cancer tissues and paracancerous tissues of LC patients (n (%)).

Pathological type	Example<br>number	SHOX2		RASSF1A		PTGER4	
		positive	negative	positive	negative	positive	negative
Cancer tissue	50	22 (44.0)	28 (56.00)	27 (54.00)	23 (46.00)	25 (50.00)	25 (50.00)
Paracancerous<br>tissue	50	45 (90.00)	5 (10.00)	48 (96.00)	2 (4.00)	41 (82.00)	9 (18.00)
X^2^ value		23.926		223.520		111.408	
P value		<0.001		<0.001		<0.001	

### Comparison of SHOX2, RASSF1A, and PTGER4 gene methylation positivity in cancer and paracancer tissues of LC patients

The methylation positivity rates of SHOX2, RASSF1A, and PTGER4 genes in cancer tissues of LC patients were 48.00%, 32.00%, and 64.00%, respectively, which were significantly higher than those in paracancerous tissues, which were 16.00%, 4.0%, and 14.00%, respectively (P < 0.05). See Table 2.

**Table 2:** Comparison of the positivity rates of detecting SHOX2, RASSF1A, and PTGER4 gene methylation in cancer tissues and paracancerous tissues of LC patients (n (%)).

Pathological<br>type	Example<br>number	SHOX2 methylation		RASSF1A methylation		PTGER4 methylation	
		positive	negative	positive	negative	positive	negative
Cancer tissue	50	24 (48.00)	26 (52.00)	16 (32.00)	34 (68.00)	32 (64.00)	18 (36.00)
Paracancerous<br>tissue	550	8 (16.00)	42 (84.00)	2 (4.00)	48 (96.00)	7 (14.00)	43 (86.00)
X^2^ value		11.765	13.279	226.272			
P value		0.001	<0.001	<0.001			

### Real-time fluorescence quantitative PCR to detect the relationship between SHOX2 methylation and clinicopathological features of LC patients

Among different pathological types and TNM stages, the highest positive rate of SHOX2 gene methylation was found in patients with adenocarcinoma and TNM stage IV LC (P < 0.05); the comparison of the positive rate of SHOX2 gene methylation among patients with different ages, genders, smoking histories or not, and patients with different histologic differentiation, maximum tumor diameter, and lymph node metastasis showed no statistically significant difference (P > 0.05). See Table 3.

**Table 3:** Relationship between SHOX2 methylation and clinicopathological features of LC patients detected by real-time fluorescence quantitative PCR (n (%))paracancerous tissues of LC patients (n (%)). Note: * is a continuous corrected chi-square test

Clinical and pathological<br>features		Example<br>number	SHOX2 gene methylation		*χ^2^* value	*P* value
			Positive<br>(n=24)	Negative<br>(n=26)		
Age (years)	<60	19	10 (41.67)	9 (34.62)	0.263	0.608
	≥60	31	14 (58.33)	17 (65.38)	1.039	0.308
Gender	male	34	18 (75.00)	16 (61.54)		
	female	16	6 (25.00)	10 (38.46)	0.120	0.729
Smoking history	have	20	9 (37.50)	11 (42.31)		
	nothing	30	15 (62.50)	15 (57.69)	6.462	0.011
Pathological type	Adenocarcinoma	11	9 (37.50)	2 (7.69)		
	Phosphorus cancer	39	15 (62.50)	24 (92.31)	2.815*	0.245
Organizational differentiation	Highly differentiated	9	3 (4.32)	6 (23.08)		
	Moderate differentiation	31	14 (58.33)	17 (65.38)		
	Low differentiation	10	7 (29.17)	3 (11.54)	0.643	0.423
Maximum tumor diameter<br>(cm)	<3	32	14 (58.33)	18 (69.23)		
	≥3	18	10 (41.67)	8 (30.77)	6.674	0.036
TNM staging	II	12	3 (12.50)	9 (34.62)		
	III	28	13 (54.17)	15 (57.69)		
	IV	10	8 (33.33)	2 (7.69)	0.675	03411
Lymph node metastasis	have	12	7 (29.17)	5 (19.23)		
	nothing	38	17 (70.83)	21 (80.77)	1.039	0.308

### Real-time fluorescence quantitative PCR to detect the relationship between RASSF1A methylation and clinicopathological features of LC patients

Among different pathological types and TNM stages, the highest positive rate of RASSF1A gene methylation was found in patients with adenocarcinoma and TNM stage IV LC (P < 0.05); the comparison of the positive rate of RASSF1A gene methylation among patients with different ages, genders, with or without smoking history, and with different histologic differentiation, maximum tumor diameter, and lymph node metastasis showed no statistically significant difference (P > 0.05). See Table 4.

**Table 4:** Relationship between RASSF1A methylation and clinicopathological features of LC patients detected by real-time fluorescence quantitative PCR (n (%)). Note: * is a continuous corrected chi-square test

Clinical and pathological<br>features		Example<br>number	RASSF1A gene methylation		*χ^2^* value	*P* value
			Positive<br>(n=16)	Negative<br>(n=34)		
Age (years)	<60	19	9 (56.25)	10 (29.41)	3.326	0.068
	≥60	31	7 (43.75)	24 (70.59)		
Gender	male	34	10 (62.50)	24 (70.59)	0.327	0.567
	female	16	6 (37.50)	10 (29.41)		
Smoking history	have	20	9 (56.25)	11 (32.35)	2.589	0.108
	nothing	30	7 (43.75)	23 (67.65)		
Pathological type	Adenocarcinoma	11	7 (43.75)	4 (11.76)	4.756*	0.029
	Phosphorus cancer	39	9 (56.25)	30 (88.24)		
Organizational differentiation	Highly differentiated	9	4 (25.00)	5 (14.71)	0.784*	0.676
	Moderate differentiation	31	9 (56.25)	22 (64.71)		
	Low differentiation	10	3 (18.75)	7 (20.59)		
Maximum tumor diameter<br>(cm)	<3	32	10 (62.50)	22 (64.71)	0.023	0.880
	≥3	18	6 (37.50)	12 (35.29)		
TNM staging	II	12	3 (18.75)	9 (26.47)	8.344*	0.015
	III	28	6 (37.50)	22 (64.71)		
	IV	10	7 (43.75)	3 (8.82)		
Lymph node metastasis	have	12	5 (31.25)	7 (20.59)	0.678	0.410
	nothing	38	11 (68.75)	27 (79.41)		

### Real-time fluorescence quantitative PCR to detect the relationship between PTGER4 methylation and clinicopathological features of LC patients

Among different pathologic types, LC patients with TNM stage IV had the highest positive rate of PTGER4 gene methylation (P < 0.05); the positive rate of PTGER4 gene methylation was comparedamong different ages, genders, with or without smoking history, and among LC patients with different pathologic types, tissue differentiation, maximum tumor diameter, and lymph node metastasis, and the difference was not statistically significant (P > 0.05). See Table 5.

**Table 5:** Relationship between PTGER4 methylation and clinicopathological features of LC patients detected by real-time fluorescence quantitative PCR (n (%)) Note: * is a continuous corrected chi-square test

Clinical and pathological<br>features		Example<br>number	PTGER4 gene methylation		*χ^2^* value	*P* value
			Positive (n=32)	Negative (n=18)		
Age (years)	<60	19	10 (31.25)	9 (50.00)	1.719	0.190
	≥60	31	22 (68.75)	9 (50.00)		
Gender	male	34	19 (59.38)	15 (83.33)	3.039	0.081
	female	16	13 (40.62)	3 (16.67)		
Smoking history	have	20	12 (37.50)	8 (44.44)	0.231	0.630
	nothing	30	20 (62.50)	10 (55.56)		
Pathological type	Adenocarcinoma	11	7 (21.88)	4 (22.22)	0.107*	0.744
	Phosphorus cancer	39	25 (78.12)	14 (77.78)		
Organizational<br>differentiation	Highly differentiated	9	4 (12.50)	5 (27.78)	1.838*	0.399
	Moderate differentiation	31	21 (65.62)	10 (55.56)		
	Low differentiation	10	7 (21.88)	3 (16.67)		
Maximum tumor diameter<br>(cm)	<3	32	18 (56.25)	14 (77.78)	2.317	0.128
	≥3	18	14 (43.75)	4 (22.22)		
TNM staging	II	12	3 (9.38)	9 (50.00)	16.105*	0.005
	III	28	8 (25.00)	7 (38.89)		
	IV	10	21 (65.62)	2 (11.11)		
Lymphatic metastasis	have	12	7 (21.88)	5 (27.78)	0.220	0.639
	nothing	38	25 (78.12)	13 (72.22)		

## Discussion

LC is a malignant tumor originating in the bronchial mucosa or glands of the lungs, characterized by atypical early symptoms and familial aggregation and genetic susceptibility, and its development is a complex biological process influenced by multisteps and multi-factors, which is related to changes in epigenetics and genetic information [11]. It has been found that DNA methylation occurs in the CpG island of the gene promoter region, which plays an important role in regulating tumor gene expression and cell differentiation in the early stages of tumors; hypermethylation of the promoter region of the CpG island of oncogenes can lead to gene silencing, and genomewide hypomethylation of proto-oncogenes leads to the aberrant activation of oncogenes, which puts the chromosomes in a precarious state, and causes the onset of tumors [12]. Therefore, the detection of LC-related proto-oncogene or oncogene expression and the alteration of its methylation status is expected to provide a molecular level basis for the early screening and diagnosis of LC. In this study, we focused on analyzing the relationship between SHOX2, RASSF1A, PTGER4 expression and their methylation and clinicopathological features of LC patients.

The results of this study showed that the positive expression rates of SHOX2, RASSF1A and PTGER4 in cancer tissues of LC patients were significantly lower than that in paracancerous tissues, suggesting that low expression of SHOX2, RASSF1A and PTGER4 is common in LC patients. The reason for the low expression of SHOX2, RASSF1A and PTGER4 in LC patients is that the development of LC is closely related to the accumulation of multi-gene variants, and the activation of proto-oncogenes promotes the proliferation and growth of cancer cells, which plays an important role in the mechanism of carcinogenesis, and the activation of proto-oncogenes promotes the proliferation and growth of cancer cells, and plays an important role in the mechanism of carcinogenesis. function, and RASSF1A, located on chromosome 3p21.3, is involved in cell cycle regulation and cell adhesion, motility and apoptosis by acting on Ras protein-related cell signal transduction pathways [13] [14]. PTGER4 is an early growth response factor-regulated gene located on chromosome 5p13.1, and the activation of this gene promotes the growth of cancer cells, which is often applied as a specific indicator for the early detection of cancer [15]. Li et al. [16] and others found that the expression of RASSF1A in multiple myeloma tissues was significantly higher than that in normal bone marrow tissues. All of these results are similar to the results of this study, suggesting that clinical attention should be paid to the expression of SHOX2, RASSF1A and PTGER4 in patients with high risk of LC, and early diagnosis and treatment should be carried out in order to improve the long-term survival rate of patients.

The results of this study showed that the methylation positivity rates of SHOX2, RASSF1A and PTGER4 genes in cancer tissues of LC patients were 48.00%, 32.00% and 64.00%, respectively, which were significantly higher than those in paracancerous tissues (16.00%, 4.0% and 14.00%), indicating that the methylation positivity rates of SHOX2, RASSF1A and PTGER4 genes were higher in cancer tissues of LC patients. DNA methylation is an epigenetic modification with the function of regulating the activation or silencing of gene transcription, and the aberrant methylation of DNA occurring in cancer tissues has been confirmed by a large number of studies [17]. Gene inactivation caused by oncogene promoter hypermethylation is an early event in cancer development, and oncogene promoter CpG island hypermethylation plays an important role in both LC pathogenesis and disease progression, as well as an important mechanism leading to oncogene inactivation [18]. Lu et al. [19] found that SHOX2 and RASSF1A, both located on human chromosome 3, are common amplification sites for LC, and their gene methylation has been used as LC diagnostic-specific biomarkers in a variety of clinical specimens, which is basically in line with the results of the present study, confirming that the methylation of SHOX2, RASSF1A and PTGER4 genes contributes to the early determination of LC, and can be used as an existing adjunctive assessment tool to conventional diagnostic methods.

The results of this study showed that among different pathological types and TNM staging, the methylation positivity rates of SHOX2 and RASSF1A genes were highest in adenocarcinoma and LC patients with TNM stage IV, and the methylation positivity rate of PTGER4 gene was highest in LC patients with TNM stage IV, which indicated that the methylation positivity rates of SHOX2 and RASSF1A genes in LC patients were related to their pathological types and TNM staging, while PTGER4 gene methylation positivity was only associated with TNM staging. Analyzing the reasons, SHOX2 and RASSF1A are more intensively studied oncogenes, whose gene methylation has a greater impact on the downstream gene products, and can contribute to cell carcinogenesis by affecting the expression of related proteins. Adenocarcinomas are rich in blood vessels, which usually show local infiltration and haematogenous metastasis earlier, accelerating cancer progression and causing hypermethylation of SHOX2 and RASSF1A, which inhibit cancer cell growth [20]. TNM staging is inextricably linked to tumour formation, progression and regression, with stage IV patients having the most advanced disease, resulting in hypermethylation of the promoter regions of oncogenes such as SHOX2 and RASSF1A and hypomethylation of the proto-oncogene PTGER4 gene [21]. Wang et al. [17] found that the positive rate of SHOX2 gene methylation in LC patients was related to pathological type and TNM stage, in which the higher the adenocarcinoma and TNM stage, the higher the methylation positivity rate, which was basically consistent with the results of the present study, confirming that there is a correlation between the positive detection rate of SHOX2, RASSF1A and PTGER4 gene methylation and the clinicopathological features of LC patients. This suggests that SHOX2, RASSF1A and PTGER4 gene methylation can be considered as a minimally invasive molecular test for assessing the clinicopathological characteristics of LC patients.

In summary, the low expression and high methylation of SHOX2, RASSF1A, and PTGER4 were prevalent in LC patients, in which the methylation positivity rates of SHOX2 and RASSF1A genes were related to their pathological types and TNM staging conditions, while the methylation positivity rate of the PTGER4 gene was only related to the pathological types.

## Dodatak

### Conflict of interest statement

All the authors declare that they have no conflict of interest in this work.
